# The impact of coronavirus disease 2019 (COVID-19) pandemic experiences on attitudes towards vaccinations: on the social, cultural and political determinants of preferred vaccination organization models in Poland

**DOI:** 10.1186/s12961-024-01214-7

**Published:** 2024-09-19

**Authors:** Piotr Żuk, Paweł Żuk

**Affiliations:** 1 The Helsinki Collegium for Advanced Studies, University of Helsinki, Helsinki, Finland; 2 Department of Sociology and Social Policy, Wrocław University of Economics, Wrocław, Poland; 3 The Centre for Civil Rights and Democracy Research, Wrocław, Poland

**Keywords:** Obligation to vaccinate, Financing of vaccinations, Vaccine policy, Research on new vaccines, Civil health society, Vaccine hesitancy

## Abstract

**Background:**

The article describes attitudes towards vaccinations in Poland in relation to issues such as voluntary versus compulsory vaccinations, the method of financing vaccinations, the method of organizing and carrying out vaccinations, the cognitive and educational aspect of vaccines (how to obtain knowledge about vaccines) and the preferred model of work and research on new vaccines. Taking into account these issues, the authors have created four ideal models of preferred vaccination policies: (a) the market model; (b) the state model; (c) the vaccine hesitancy model; and (d) the civic–social model. This perspective makes it possible to better understand and learn about the various motives behind the attitudes of anti-vaccination movements, as well as to notice cracks and divisions among vaccination supporters and their attitudes towards the financing and organization of vaccinations.

**Methods:**

The study was carried out using the CATI method on a representative random-quota sample of Polish society of 1000 people aged 18 and over. The study took age, sex, education and the size of the place of residence into account.

Additionally, in the Socio-demographic factors influencing attitudes towards vaccination practices in Poland section, we used the chi-squared test and regression analysis of factors influencing vaccination practices in Poland. PASW Statistics 18 (a version of SPSS) software was used for statistical analysis. Significant correlations were demonstrated at a significance level of 0.05% Pearson.

**Results:**

This article has shown that attitudes towards vaccinations are embedded in broader divisions and orientations related to the vision of the social order: the role of the state, the organization of healthcare and payments for vaccinations and medical services, as well as preferred ways of knowledge production in society and work on new vaccines. The political sympathies and the age of the respondents were the most important variables influencing vaccination behaviour. The education of the respondents was less important.

**Conclusions:**

A few years after the peak of the pandemic, the scope of anti-vaccination attitudes in Polish society ranges from 20% of the population (dogmatic anti-vaxxers) to 30% (vaccine hesitancy occurring depending on attitudes towards vaccinations).

## Introduction

Data and analyses show that the mass vaccination campaign saved millions of potential victims of the coronavirus disease 2019 (COVID-19) pandemic [1]. However, medical facts do not generally translate into social attitudes and opinions. Analyses on vaccine hesitancy and opposition sought to uncover the various factors that influenced attitudes towards vaccinations (level of trust, reliance on social media, education, income, and ultimately, overall attitudes towards scientific knowledge and vaccinations) [2]. However, these analyses very often only refer to the psychological [3] and personality variables of an individual, completely omitting the perspective and logic of the social system and the framework of health policy [4]. In our article, we change the perspective of the analysis. We address vaccination policy more comprehensively and go beyond simple binary opposition: supporters versus opponents of vaccination. We believe that the dispute over the attitude towards vaccinations and their organization can be treated as an indicator of more general and ideological disputes in society about the model for organizing public life and the vision of a ‘good and desirable’ social order. In these social disputes and conflicts, there is, of course, room for conflicting opinions about the desired model of healthcare and the organization of vaccination policy. At the most general level, however, it is part of the disputes about the economic model of society, the attitude towards the market and the role of the state, as well as the attitude towards the independent sphere of civil society. In the background, there are also conflicts about the role and importance of the traditional family in raising children, the rights of an individual in the context of their relationship with the institutional macro-social level and the attitude towards medical and pharmaceutical corporations. This perspective allows us to better understand and learn about the various motives behind the attitudes of anti-vaccination movements, as well as notice the cracks and divisions among vaccination supporters and their attitudes towards the financing and organization of vaccinations, compulsory and voluntary vaccinations and the preferred methods of conducting and supervising research on new vaccines. Our considerations and reflections are based on empirical material from survey research carried out using the computer-assisted telephone interviewing (CATI) method on a representative sample of Polish society. However, we assume that similar models can be analysed and studied in any other society. From such a perspective, the attitude towards vaccinations proves to be an element of a more complex conflict over the desired social model, the preferred model of knowledge and supervision over the social production of knowledge and medical practice.

The four models of attitudes towards vaccination policy proposed in this article should be treated as certain ideal types as understood by Max Weber: these models do not have to occur in their pure form in the real social world, but allow us to better understand and explain this world. To put it differently, ideal types in the Weberian sense are certain conceptual constructs that make it easier to understand reality but do not necessarily constitute its direct reflection: “It has the significance of a purely ideal limiting concept with which the real situation or action is compared and surveyed for the explication of certain of its significant components” [5]. The four ideal models of attitudes towards vaccinations distinguished in this article are: (a) the market model; (b) the state model; (c) the vaccine hesitancy model (which can also be called the traditionalist-anti-modernization model in our analyses); and (d) the civic–social model. These models are described and explained in detail in Sect. 3. Here we will only point out that they are related not only to the vision of “good healthcare”, but also to the more general desired vision of “the right social and political order”.

These four models translate into the political preferences of respondents, and can even be said to be among the indicators of political sympathies, and at the same time, an element of building and reproducing political identities. Of course, this process also works the other way around: specific political sympathies shape and strengthen particular attitudes towards vaccinations.

Our findings may be useful not only for subsequent analyses and social research on vaccinations, but also for helping reform the organization of vaccinations and planning vaccination policies in various social environments. They may also be used in the debate on the process of democratization of healthcare.

## Social and medical context and rate of vaccination in Poland during the pandemic

Polish society is a good subject for analysing vaccination policy preferences after the experience of the COVID-19 pandemic because the coronavirus mortality rate in Poland was exceptionally high compared with the general trends in Europe. In the first phase of the pandemic, Poland had significantly fewer COVID-19 deaths per million than the average in the European Union (EU) because of a very restrictive approach to the pandemic (total lockdown and many social restrictions). Later, however, the situation gradually worsened. A direct link between high mortality and low vaccination rates has been repeatedly demonstrated. According to Kundzewicz et al.:

One of the likely reasons why the numbers of COVID-19 deaths and all-cause deaths per million in Poland were much higher than the EU average was the low national vaccination rate in the country. Most Polish COVID-19 deaths since 2021 were in the unvaccinated. By March 22, 2022, only 59.06% of Poles were fully vaccinated and only 30.76% boosted, far below EU average values of 72.95% and 49.56%, respectively. The full vaccination rate of seniors (categories 60+ and 70+), who were most vulnerable, was below 80% [6] (p. 3).

It can be said that, in general, in the countries of Central and Eastern Europe, a similar low vaccination rate and an accompanying high mortality rate per million inhabitants were observed. In Bulgaria, the vaccination rate was the lowest and amounted to 29.70%, and the number of deaths per million inhabitants was 5271. In Czechia, it was 63.93% and 3675 deaths per million; in Slovakia, 50.68% and 3507; in Romania, 42.25% and 3385; and in Hungary 64.19% and 4686, respectively [7]. Analyses indicate that the top 10 vaccine-sceptic populations in the EU were in the eastern part of the continent. This is explained by “distrust of national governments and medical professionals as sources of relevant information in Eastern Europe that are relevant” [8] (p. 3186).

In Poland, in addition to the low vaccination rate, there were also other reasons for the high mortality rate during the pandemic. On the one hand, there was an insufficient number of doctors and medical workers, many of whom migrated to Western European countries after Poland joined the EU [9]. On the other hand, there were cultural and ideological disputes over vaccinations in Poland long before the pandemic [10]. In Poland, numerous conspiracy theories and aggressive anti-vaccination and misinformation-spreading comments could be found on the internet and in social media during the pandemic [11].

During the pandemic, ethical doubts about vaccinations were also reinforced by the Catholic Church, which claimed that the cell lines of aborted foetuses were used to produce the vaccines. In December 2020, the Expert Team of the Polish Bishops Conference on Bioethics stated in its position on vaccines:

Moral responsibility is borne by anyone who uses the cells or tissues of an aborted human foetus (and manipulates such parts) in specific processes where abortion is an integral element in achieving a specific goal (here, producing a vaccine). In this attitude, by participating in the production of the vaccine, the person expresses their acceptance of abortion, and therefore of moral evil [12].

Additionally, in October and November 2020, there were mass demonstrations against the right-wing populist government, which completely banned abortion through the decision of the Constitutional Tribunal (controlled by right-wing populists). At the peak of the demonstrations at the turn of October and November 2020, there were 600,000 people on the streets of Polish cities [13]. These mass protests completely suspended social distancing rules. As was pointed out, large gatherings may have a widespread impact on the transmission of COVID-19, and even fully vaccinated people should maintain hygiene and caution in social distancing [14].

## Ideal types of vaccination policy and organization at the macro-social level

Attitudes towards vaccinations may be influenced by ideological issues and cultural attitudes, political and economic views and many other social factors related to the preferred model of organizing social life. Thus, it is worth checking the possible configurations of all these factors towards vaccination policy. On the basis of the literature and our observations, we propose four types of ideal models (preferred orientations and attitudes) of vaccinations on the basis of issues such as: voluntary versus compulsory vaccinations, the method of financing vaccinations, the method of organizing and carrying out vaccinations, the cognitive and educational aspect of vaccines (how to obtain knowledge about vaccines) and the preferred model of work and research on new vaccines.

### The market model

This model includes all the attitudes, ideas and suggestions regarding the organization of vaccinations, which come down to the belief that market logic should be the main driving force for the production of new vaccines and vaccination policies. In this approach, both the medical and pharmaceutical markets cover all stages of this activity, from product development to manufacturing and marketing [15]. From this perspective, market mechanisms would also ensure the development of research on new vaccines and access to them in poor countries. The idea of advance purchase commitments may serve as an illustration of this position:

One proposal to incentivize private sector R&D investments in products for diseases concentrated in poor countries is for sponsors (rich-country governments, private foundations, or international organizations such as the World Bank) to undertake “advance purchase commitments” for desired products, such as an HIV vaccine. A commitment to purchase these products in advance of their development would create market incentives for firms to develop needed vaccines [16] (p. 67).

Faith in market mechanisms comes down to reducing vaccine policy to the principles of political economy, in which the laws of supply and demand are the main forces driving both international and domestic vaccine producers. A market shock caused by, for example, a pandemic, is a sufficient factor for introducing new products [17]. Here, the same methodological reductionism applies to human behaviour: concepts such as “public good”, “social relations” or “social context” are not taken into account, but all human behaviour is reduced to the choice of the individual. A strong assumption is made about the rational choice of the individual who, guided by maximizing their benefits (including avoiding the disease), will at some point decide to be vaccinated due to the influence of a large percentage of the infected population [18]. Although some market representatives see a contradiction between the profitability of vaccine production and its widespread availability, the prevailing belief is that “prizes, market subsidies, grants, and tax incentives” [19] may make vaccines profitable and broadly accessible [19].

The main risks of this model are related to the marketization of vaccination policy and leaving public health in the hands of anonymous market forces. In practice, this reduces the responsibility of public entities and public policy for public health, health education, vaccination levels and the effects of potential pandemics. In this variant, human health is treated like any other product for sale, and not as a special social value that should be protected regardless of the class and economic position of individuals.

### The state model

Regardless of whether it is a welfare or liberal state, we assume that one of a state’s basic duties is to protect against threats to both individual and social lives. In the bureaucratic-state model, the protection of health and life is one of the basic duties of public authorities. This means that:

The state has the responsibility to protect public health and societal life against dangerous infections. This responsibility involves creating conditions in which people can live together safely, in particular by preventing people from (unintentionally) harming one another but also protecting against potentially dangerous infection risks in an environment that may affect large groups of people [20] (p. 7165).

This assumption implies that the state is obliged to finance healthcare and provide free access to the achievements of modern medicine:

The state has moral reasons to offer equal access to … vaccination – of course within the limits of reasonable healthcare expenditures. In some cases, preventive vaccination may be closely linked to patient care. Given that rotavirus infections seem to be especially risky for specific patient groups, vaccination should be considered as part and parcel of basic healthcare for these groups [20] (p. 7165).

This philosophy of medical policy implies that vaccination policies yield collective as well as individual benefits and that the state has responsibilities in regard to both [21]. The state-model approach to vaccination policy is associated with a broader perspective on public health, in which the state has an active role to play. The government in this approach has to find the right balance between individual interests and the public interest of the entire society [22]. However, the weakness of the state model is that it transfers all responsibility for the health system and vaccination policy to the state, an anonymous bureaucratic system and impersonal mechanism for making decisions about the health of the entire population. The desire for total control over social behaviour within the territory of the state for health reasons refers to Foucault’s classic concept of biopolitics. From this perspective, control over the human body and health is embedded in the broader context of state policy [23]. During the COVID-19 pandemic, some commentators noticed a disturbing combination of state and police violence with epidemiology and state management. These experiences were associated with Foucault’s biopolitics [24]. However, the diminished role of social organizations, the small importance of independent civic initiatives in the area of health policy, the collapse of grassroots social activity and the passive attitude of individuals (who automatically accept all state recommendations) towards health challenges are the greatest weaknesses of the state model.

### The vaccine hesitancy model

Although the literature distinguishes between hesitancy and rejection [25], in this article we include these two attitudes in the same category of people who are hesitant about vaccinations to a greater or lesser extent. Studies on attitudes towards vaccinations contain the opinion that in the entire spectrum of possible orientations (from full acceptance to complete refusal of vaccinations), the vaccine hesitant individual is in the middle of this continuum: “Vaccine-hesitant individuals may refuse some vaccines, but agree to others; they may delay vaccines or accept vaccines according to the recommended schedule, but be unsure in doing so” [26] (p. 1764). However, the attitudes of hesitancy and rejection are distinguished because they may have the same effect (avoiding vaccination), but the reasons for and the justification of hesitancy as well as the possibilities of changing this attitude are different. After all, it is more important for us to determine what views on health policy and ideas on vaccination policy go hand in hand with the views included in the term “hesitancy”.

Reactions to previous pandemics have shown that large cultural differences influence public responses to the threat [27]. Generally, it is emphasized that “acceptance of vaccination is an outcome behaviour resulting from a complex decision-making process that can be potentially influenced by a wide range of factors” [28]. As these factors are complex, the role of social sciences is irreplaceable in identifying the main barriers and determining the reasons for the lack of acceptance of vaccinations (which may be different in different groups, classes and social contexts). It is also crucial in developing appropriate procedures for the health service, especially since hesitancy is not a new attitude that appeared with the COVID-19 pandemic but occurred much earlier [29]. Hesitancy has existed in society since the beginning of vaccinations. In the nineteenth century, the main arguments raised against vaccination in England varied by social class: while the middle class saw vaccination as an intervention of the state upon sacred individual liberty, the working-class anti-vaccination movement interpreted the compulsory vaccination as a tyrannical appropriation of working bodies via legislation [30]. During one of the last smallpox epidemics in Europe, which broke out in Wrocław in 1963, a “black market” of false vaccination certificates was created for those who did not want to take mandatory vaccines [31]. Each time, vaccine hesitancy influences vaccine uptake [32]. A dominant public discourse on vaccinations, the related risks and trust in science as such in a given society significantly affect attitudes within the vaccine hesitancy model [33].

Vaccination opponents are against compulsory vaccinations and this aspect is the most spectacular from the perspective of the media and public opinion. However, people with the hesitant orientation are also sceptical of the state’s involvement in work on new vaccines and ensuring free access to vaccines (also through free vaccinations). Moreover, the literature on the subject tries to explain the attitudes of people hesitant about vaccinations with their postmodern cultural orientation (which treats personalized truths presented online as legitimate and assumes that the number of truths can be infinite and does not require any verification procedures [34]). In social practice, however, supporters of “niche truths” recognize them as the only true ones. In this sense, supporters of “niche truths” and the right to an apparent choice of “many equal truths” take the roles of religious fundamentalists, who recognize only “one holy truth”. Incidentally, they reject the findings of modern medicine and science. Thus, the greatest danger in the hesitancy model is that the most absurd conspiracy theory can be treated as equal to knowledge created in the process of long research and experiments. In this context, it is worth remembering that medicine must be modern-sceptical, evidence-based and self-critical, and at the same time, open to the voice of civil society [35]. However, this should not be the voice of the sectarian anti-vaccination groups, but of grassroots social initiatives that actually work for the socialization of health care and greater public participation in health policy planning. Increasing their role in public health is associated with the fourth model: the civic–social model.

### The civic–social model

However, scepticism of the state does not always have to go hand in hand with the vaccine hesitancy model. Public health services do not have to be organized only by the state administration. Many other actors in the public sphere engage in public health services outside the state structures. As Angus Dawson and Marcel Verweij write:

For example, many of the most promising health-promoting activities take place on a local level, in communities, schools, places of work, worship or neighbourhoods, where legal regulations and the role of government is small or non-existent [36] (p. 1).

Various types of non-governmental organizations, associations, foundations, social movements for the promotion of public health and social and civic research institutes can be added to this list. The same non-state actors can also advocate for vaccination policy and the dissemination of vaccines in society. This is particularly important in countries with low vaccination rates, financial barriers, a weak public health service network and geographical areas without professional healthcare [37]. Additionally, in Western societies, civil society actors have many functions to fulfil. Namely, civil society organizations can provide a form of pressure on political decision-makers, introduce new ideas about the benefits of vaccinations to the public sphere and also be a bridge for building relationships and information channels between scientific centres and various social groups [38]. As regards energy transition, civil energy society can compensate for neglect and deficiencies on the part of the state, as well as stimulate grassroots social activity for the development of renewable energy [39]. The health civil society can play the same role in modernizing and democratizing healthcare. As reported in *The Lancet*, civil society organizations play a growing role in solving global health challenges because they provide “essential voices in a discordant global health conversation often dominated by risk-adverse multilaterals, corrupt governments and neo-colonial donors” [40]. Similarly, in the case of vaccinations and healthcare, we can also talk about the phenomenon of a “civil health society”. Grassroots citizen action always strengthens the democratic nature of decision-making processes, and even if it diminishes the power of government officials, it can boost the democratic legitimacy of vaccination policy [41]. Moreover, the participation of civil organizations in shaping vaccination policy and organization in the areas of civic education, vaccination campaigns and the policy for research on new vaccines may seriously weaken the influence of anti-vaccination movements, which tend to present themselves as the “voice of the people” and “ordinary people” against the state and large corporations. For now, the greatest weakness of social organizations working for better healthcare and the popularization of vaccinations is their low importance and inability to get their demands across to the mainstream of health policy. T​his is especially true for countries such as Poland and other Eastern European societies with a weak tradition of strong civic movements [42, 43].

## Methods

The study was carried out using the CATI method on a representative random-quota sample of Polish society of 1000 people aged 18 years and over. The study recorded age, sex, education and the size of the place of residence. A database of mobile phone numbers was used to create the research sample.

The statistical analysis of the results involved frequency analysis, descriptive statistics and cross-tabulations. Additionally, in the Socio-demographic factors influencing attitudes towards vaccination practices in Poland section, we used the chi-squared test and regression analysis of factors influencing vaccination practices in Poland. PASW Statistics 18 (a version of SPSS) software was used for the statistical analysis. Significant correlations were demonstrated at a significance level of 0.05% Pearson. These significances are based on a test of proportion corrected for demographic weight and statistics. The results presented in bold in the tables are statistically significant. While the results in individual columns add up to 100%, the results in individual cells have been rounded to full digits (without decimal places).

To ensure the reliability of our research, we followed Charles Wright Mills’ principle (with which we agree) to be guided primarily by the “sociological imagination” in the analyses of social sciences. This makes it possible to describe and translate individuals’ dilemmas and choices into the language of systemic problems [44]. This is also a good principle in the research and analysis of health behaviours. The behaviours of individual people in the health sphere always take place within the framework and contexts of actually existing public health systems. At the same time, Mills warned against falling into the trap of “abstract empiricism”, which involves emphasizing the importance of numbers and methodological procedures, and ignoring real social problems and important mechanisms influencing the attitudes of individual groups and social classes [44]. Research resulting in public health articles and analyses must be communicable and understandable also to people outside academic circles, who do not use statistical jargon. We also direct our article outside academic circles, to people, social organizations and all collective entities interested in the challenges of vaccination policy. For us, the results of quantitative research are only a certain illustration of the social trends being described, and we certainly avoid fetishizing numbers and statistical tests.

We also have a strong opinion about the CATI method we used. Various survey reports have indicated that the CATI method gives the most reliable results under Polish conditions [45]. This is probably because, on the one hand, the low level of social trust in Poland means that in face-to-face surveys, respondents do not always give true answers in line with their beliefs. CATI surveys give a sense of greater anonymity and the so-called interviewer effect (the interviewer influences the respondent’s statements) is smaller. On the other hand, CAWI surveys (online surveys) are by definition unrepresentative in Poland because some members of selected social groups (e.g. seniors) are digitally excluded and do not use the internet. These facts briefly explain why we decided to use the CATI method. Of course, CATI also has its drawbacks. One of them is the limited length of an interview. Too many questions cannot be asked here because, unlike in “face-to-face surveys, respondents may become bored more quickly and end the conversation”. In our research, we only present results from full interviews. Excluding some answers from the analysis results reduces the sample size and disrupts the randomness of the sample. However, if we try to distribute the percentage of the “don’t know/hard to say” category and the “refused to answer” category, we may obtain false results.

In addition to assessing the vaccination campaign during the COVID-19 pandemic, our research results show how preferences regarding the vaccination organization model were distributed in society. On the basis of social attitudes towards the issue of voluntary versus compulsory vaccinations, the method of financing vaccinations, the organization and implementation of vaccinations, and educational and research policy, four ideal models have been created and are described in Sect. 3. Each model had its indicators in each of the examined dimensions. Table 1 contains the models’ descriptions.

**Table 1:** Models of attitudes towards vaccinations and health policy

	Market model	State model	Vaccine hesitancy model	Civic–social model
Organization of vaccinations	Market and interested consumers	Public health service institutions	No one should have the right to organize mass vaccinations	Local government, municipal and school institutions
Legal aspect of and voluntary access to vaccinations	Full and voluntary access to vaccinations at every stage of life	Compulsory up to 18 years of age and in all age categories during epidemics	There is no obligation to vaccinate, and parents have the right to refuse to vaccinate their children in public institutions (schools, hospitals)	Obligation to vaccinate children up to 18 years of age against infectious diseases. Later in life, vaccinations are voluntary
Cognitive and educational aspect	Everyone on their own acquires information on the market of ideas and expands their knowledge about the advantages and threats of vaccinations	The state and its structures determine the scope of vaccinations and shape the model of public health knowledge	Parents decide about their children’s education and neither the school nor the state can impose vaccinations	Civic research and scientific institutions, social organizations working for public health and civil society actors disseminate health education and verify and supplement state policy in this area
Financial aspect	Vaccinations are paid by the individuals concerned	Vaccinations for children and adolescents up to 18 years of age are free and paid for adults (except for vaccinations for selected diseases during pandemics)	Vaccinations are paid and are not widely available	All vaccinations are free and financed by health contributions paid by all citizens
Technological and development aspect	Private companies and concerns should work on new vaccines and develop new treatment methods	The state and its institutions should fully control the work on new vaccines and the development of treatment methods	Neither the state nor private companies should have any influence on the development of human treatment methods	The activity of the state and private companies should be supplemented by civil society actors and social organizations should be able to supervise the goals and methods of activities of the state and market institutions in the area of health policy

## Results and discussion

### Assessment of vaccinations during the COVID-19 pandemic and socio-political factors

The research results obtained confirm previous analyses that have indicated the importance of political sympathies in shaping attitudes towards vaccinations. Political identification resulted in significant differences in the approach to vaccination between Democrats and Republicans in the United States during the flu season of 2009 and 2010 [46]. In Poland, both before and during the COVID-19 pandemic, anti-vaccine attitudes were strongly connected with extreme and populist right-wing circles [47]. How did these political variables influence the assessment of the vaccination campaign and what other factors determined the assessment of the organization of vaccination against the coronavirus a few years later?

Have vaccinations saved many people from losing their health and lives? The answer seems obvious, but how did Poles assess this issue? Of the sample surveyed, which was representative of the overall Polish population, 53% answered this question positively, 22% negatively and as many as 25% had no clear opinion on this subject (Table 2). What factors contributed to a higher positive assessment of the vaccination campaign than the average for the entire society? First of all, the age of the respondents: among people over 60 years of age, as many as 64% had a positive opinion about vaccinations. Another factor was the respondents’ higher education (58%), and above all, their political sympathies. The most positive opinions about vaccinations were expressed by the liberal electorate of the Civic Coalition (72%) (the once neoliberal party has turned into a social-liberal one) and left-wing sympathizers (71%). The vaccination campaign was also highly assessed by those who were vaccinated against COVID-19 at least twice (72%). Generally, it can be said that respondents vaccinated with a basic dose and at least one booster positively assessed the vaccination campaign and consistently presented a pro-vaccine attitude in answers to the other questions.

**Table 2:** A few years after the outbreak of the pandemic and the widespread vaccination campaign against COVID-19, we have more knowledge on this subject. Do you agree with the following statement: ‘Vaccinations have saved many people from losing their health and lives’? (In percentage)

	Sex			Age						Education			
	Total	Male	Female	18–24	25–29	30–39	40–49	50–59	60+	Primary or middle	Vocational	Secondary	Higher
%													
(1) Definitely not	12	**16+ **	**7−**	11	13	13	**23+ **	9	**6−**	20	11	10	10
(2) Somewhat not	11	11	10	14	19	**17+ **	10	10	**5−**	**2−**	10	13	13
(3) Hard to say	25	**21−**	**28+ **	19	**13−**	23	30	27	25	29	29	24	**19−**
(4) Somewhat yes	26	23	29	24	27	27	19	23	31	29	24	26	25
(5) Definitely yes	27	29	26	32	29	**21−**	**18−**	32	**34+ **	21	26	27	**33+ **
(1 + 2) NO	22	**28+ **	**17−**	24	31	**29+ **	**33+ **	19	**10−**	22	20	23	23
(4 + 5) YES	53	52	54	56	55	48	**37−**	54	**64+ **	50	50	53	58
Total	100	100	100	100	100	100	100	100	100	100	100	100	100
Mean	3.47	3.37	3.56	3.53	3.40	3.27	2.99	3.59	3.83	3.29	3.45	3.46	3.59

	Political sympathies									How often do you attend masses, church services or religious meetings?						
	Law and Justice	The Third Way: Poland Szymon Hołownia’s Poland 2050 and Polish People’s Party	Civic Coalition	New Left	Confederation	Nonpartisan Local Government Activists	There is One Poland	Other	I don’t know, it’s hard to say	Every day or several times a week	Once a week every Sunday	Once or twice a month	Once or twice a year	Less than once a year	Never	Don’t know, I refuse to answer
%																
(1) Definitely not	10	**5−**	**3−**	6	**33+ **	12	**63+ **	29	9	15	13	13	13	**2−**	12	6
(2) Somewhat not	7	9	9	10	**22+ **	21	7	8	15	25	9	14	10	13	8	8
(3) Hard to say	25	27	**16−**	**14−**	22	33	12	21	32	31	23	25	26	23	22	39
(4) Somewhat yes	28	31	25	32	**11−**	19	16	28	26	**9−**	28	25	24	31	26	21
(5) Definitely yes	29	28	**46+ **	39	**12−**	15	**2−**	14	**17−**	20	27	23	27	32	31	26
(1 + 2) NO	17	**14−**	**12−**	16	**54+ **	34	**70+ **	37	25	**40+ **	22	27	23	15	21	14
(4 + 5) YES	57	59	**72+ **	**71+ **	**23−**	**34−**	**18−**	42	43	**29−**	55	48	51	63	57	47
Total	100	100	100	100	100	100	100	100	100	100	100	100	100	100	100	100
Mean	3.58	3.69	4.03	3.89	2.48	3.03	1.86	2.90	3.27	2.94	3.46	3.32	3.43	3.78	3.56	3.52

	Have you been vaccinated against COVID-19 (coronavirus)?			
	I have not been vaccinated	I have been vaccinated once	I have been vaccinated more than once	I don’t remember
%				
(1) Definitely not	**34+ **	10	**2−**	30
(2) Somewhat not	**21+ **	**22+ **	**5−**	35
(3) Hard to say	**34+ **	25	**21−**	19
(4) Somewhat yes	**6−**	29	**33+ **	**–**
(5) Definitely yes	**4−**	**15−**	**39+ **	17
(1 + 2) NO	**55+ **	31	**7−**	**65+ **
(4 + 5) YES	**10−**	44	**72+ **	**17−**
Total	100	100	100	100
Mean	2.25	3.17	4.01	2.39

In turn, the most negative opinions about the vaccination campaign were among the electorate of the right-wing populist parties, who received a total of approximately 11% of votes in the latest parliamentary elections. Among the supporters of the largest of these, that is, the Confederation, which is present in the parliament, 54% had a negative opinion about the vaccination campaign. Moreover, 34% of Nonpartisan Local Government Activists and 74% of There is One Poland had a negative opinion about vaccinations (Table 2). These two groups are not represented in the parliament, but it can be considered that together with the populist-right Confederation, they constituted the political representation of opponents of vaccinations.

It was not only in Poland that political views influenced vaccinations and the preferred model of vaccination policy. It was similar in the United States, where political sympathies were a key factor in shaping attitudes towards COVID-19 vaccines. Democrats were much more likely than Republicans to take the threat of the virus seriously and to support efforts to control it [48]. In general, international analyses of this problem have shown that ideological extremism matters for vaccination scepticism: The more ideologically extreme respondents are, the more likely they are to think that vaccines cause diseases against which they should protect [49]. In Poland, although the arguments of anti-vaxxers were popular in social media, the mainstream media treated anti-vaccination activists as a “noisy minority” or “lonely Robinsons Crusoes”. Locked in their islands, they close themselves off from the current discourse, forming their own knowledge and their own practices [50]. However, political and ideological polarization influences the preferred model of vaccination policy in both the United States and Poland. Misinformation is spreading through right-leaning media programmes and on social media [51].

A factor that additionally strengthened the negative assessment of vaccinations was devout religiosity. Of the people who participated in religious practices several times a week, as many as 40% did not agree with the opinion that vaccinations saved the lives of many people. This confirms that religious fundamentalism is contradictory to scientific knowledge. In Poland, there is a strong connection between the Catholic Church and right-wing conservative views [52]. Another study has shown that religious leaders (as opposed to, for example, doctors) generally do not have an effect on reducing vaccine distrust [53].

Who openly criticized the vaccination campaign, considering it to be “a waste of money”? This opinion was expressed primarily by people who had not been vaccinated (55%) and again by the electorates of right-wing populist parties, the Confederation in particular (59%). Generally speaking, 31% of people in society as a whole agreed with this anti-vaccine view (Table 3). This group of people includes both opponents of vaccinations and those with the hesitant orientation.

**Table 3:** A few years after the outbreak of the pandemic and the widespread vaccination campaign against COVID-19, we have more knowledge on this subject. Do you agree with the following statement: ‘The vaccination campaign was an unnecessary waste of money’? (In percentage)

	Sex			Age						Education			
	Total	Male	Female	18–24	25–29	30–39	40–49	50–59	60+	Primary or middle	Vocational	Secondary	Higher
%													
(1) Definitely not	30	33	27	26	**43+ **	25	26	32	33	24	**20−**	30	**41+ **
(2) Somewhat not	18	16	19	15	19	18	15	16	21	19	16	17	19
(3) Hard to say	21	21	22	22	**8−**	21	22	21	24	29	27	22	**12−**
(4) Somewhat yes	11	10	11	14	12	8	11	13	10	8	10	12	11
(5) Definitely yes	20	20	20	24	18	**29+ **	25	19	**11−**	20	26	18	17
(1 + 2) NO	48	50	46	41	**62+ **	42	41	47	**54+ **	43	**37−**	48	**60+ **
(4 + 5) YES	31	30	32	38	30	37	36	31	**22−**	28	36	30	29
Total	100	100	100	100	100	100	100	100	100	100	100	100	100
Mean	2.73	2.67	2.78	2.95	2.43	2.99	2.94	2.71	2.46	2.80	3.06	2.70	2.45

	Political sympathies									How often do you attend masses, church services or religious meetings?						
	Law and Justice	The Third Way: Poland Szymon Hołownia’s Poland 2050 and Polish People’s Party	Civic Coalition	New Left	Confederation	Nonpartisan Local Government Activists	There is One Poland	Other	I don’t know, it’s hard to say	Every day or several times a week	Once a week every Sunday	Once or twice a month	Once or twice a year	Less than once a year	Never	Don’t know, I refuse to answer
%																
(1) Definitely not	30	32	**46+ **	**56+ **	**9−**	**14−**	**8−**	**10−**	27	19	25	**21−**	36	33	**37+ **	35
(2) Somewhat not	21	**27+ **	15	11	15	16	19	40	13	16	21	21	17	17	15	11
(3) Hard to say	22	17	16	13	17	29	6	34	19	31	22	28	**15−**	22	19	24
(4) Somewhat yes	9	12	11	10	10	14	8	5	15	22	9	**6−**	14	12	11	16
(5) Definitely yes	18	**13−**	**13−**	**10−**	**49+ **	28	60	11	27	13	22	25	18	17	19	14
(1 + 2) NO	51	59	**61+ **	**67+ **	**24−**	**29−**	27	50	39	35	46	42	53	50	52	46
(4 + 5) YES	27	25	**24−**	**20−**	**59+ **	42	67	16	42	34	32	30	32	28	29	30
Total	100	100	100	100	100	100	100	100	100	100	100	100	100	100	100	100
Mean	2.63	2.47	2.29	2.06	3.76	3.27	3.92	2.66	3.03	2.93	2.82	2.92	2.62	2.62	2.59	2.62

	Have you been vaccinated against COVID-19 (coronavirus)?			
	I have not been vaccinated	I have been vaccinated once	I have been vaccinated more than once	I don’t remember
%				
(1) Definitely not	**11−**	**10−**	**40+ **	30
(2) Somewhat not	**9−**	26	**20+ **	7
(3) Hard to say	26	**32+ **	**18−**	19
(4) Somewhat yes	14	12	9	27
(5) Definitely yes	**40+ **	20	**12−**	17
(1 + 2) NO	**20−**	**36−**	**61+ **	37
(4 + 5) YES	**55+ **	31	**21−**	44
Total	100	100	100	100
Mean	3.64	3.04	2.32	2.94

More divided opinions were recorded when assessing the solution, which was promoted in Poland for a while at the turn of 2021 and 2022, although it had no clear legal basis. The employer could check whether an employee had been vaccinated. The Polish Labour Inspectorate found it “deliberate and justified for employees to be vaccinated against COVID-19 as widely as possible”, emphasizing at the same time that “under the currently applicable legal provisions, undergoing these vaccinations is voluntary” [54]. It also stated that:

… the employer is obliged to keep a register of work exposing employees to harmful biological factors classified to hazard groups 3 or 4 and a register of employees exposed to harmful biological factors classified to hazard groups 3 or 4 [54].

One of the mentioned hazard groups (i.e. group 3) includes the severe acute respiratory syndrome coronavirus 2 (SARS-COV-2) virus. Although a bill appeared in November 2021 allowing employers to verify whether employees were vaccinated against COVID-19, it was never passed. However, our research results show that as many as 50% of respondents were against introducing this solution, and 33% of the entire surveyed population supported the employer’s right to check vaccination certificates (Table 4). This idea was most appreciated by people vaccinated with at least two doses (39%) and left-wingers (50%). The topic related to safety at work in the context of the threat of viral diseases and the obligation to have a vaccination certificate requires further analysis.

**Table 4:** A few years after the outbreak of the pandemic and the widespread vaccination campaign against COVID-19, we have more knowledge on this subject. Do you agree with the following statement: ‘Vaccination certificates should not be demanded in the workplace’? (In percentage)

	Sex			Age						Education			
	Total	Male	Female	18–24	25–29	30–39	40–49	50–59	60+	Primary or middle	Vocational	Secondary	Higher
%													
(1) Definitely not	17	**20+ **	**14−**	21	18	**7−**	**27+ **	18	14	19	18	16	15
(2) Somewhat not	16	**13−**	**20+ **	16	19	13	**8−**	20	**22+ **	23	14	16	16
(3) Hard to say	17	16	18	11	13	**11−**	**6−**	18	**30+ **	28	23	**13−**	**13−**
(4) Somewhat yes	14	**11−**	**16+ **	10	15	14	11	15	15	13	13	14	14
(5) Definitely not	36	**40+ **	**32−**	42	35	**54+ **	**48+ **	29	**19−**	**17−**	32	**41+ **	**42+ **
(1 + 2) NO	33	33	33	37	36	**21−**	35	37	36	42	32	32	31
(4 + 5) YES	50	51	48	52	50	**68+ **	**59+ **	45	**34−**	**30−**	45	**55+ **	**57+ **
Total	100	100	100	100	100	100	100	100	100	100	100	100	100
Mean	3.35	3.39	3.33	3.35	3.32	3.94	3.44	3.19	3.03	2.87	3.28	3.47	3.53

	Political sympathies									How often do you attend masses, church services or religious meetings?						
	Law and Justice	The Third Way: Poland Szymon Hołownia’s Poland 2050 and Polish People’s Party	Civic Coalition	New Left	Confederation	Nonpartisan Local Government Activists	There is One Poland	Other	I don’t know, it’s hard to say	Every day or several times a week	Once a week every Sunday	Once or twice a month	Once or twice a year	Less than once a year	Never	Don’t know, I refuse to answer
%																
(1) Definitely not	14	**9−**	**22+ **	21	**28+ **	19	6	6	**8−**	20	**10−**	16	**24+ **	**31+ **	16	8
(2) Somewhat not	21	17	13	**30+ **	**2−**	**3−**	**–**	11	13	21	13	14	17	18	19	29
(3) Hard to say	16	24	21	16	**5−**	**4−**	**–**	10	21	21	22	16	**10−**	15	19	22
(4) Somewhat yes	15	**23+ **	15	13	**5−**	17	12	17	11	**3−**	18	20	10	11	11	9
(5) Definitely not	34	**28−**	**30−**	**21−**	**60+ **	56	**82+ **	56	47	35	37	34	40	26	36	32
(1 + 2) NO	35	26	35	**50+ **	30	22	**6−**	17	**20−**	41	**23−**	30	41	**48+ **	34	37
(4 + 5) YES	50	50	**44−**	**34−**	**65+ **	**74+ **	**94+ **	73	59	38	55	54	50	37	47	41
Total	100	100	100	100	100	100	100	100	100	100	100	100	100	100	100	100
Mean	3.35	3.43	3.17	2.84	3.67	3.88	4.63	4.06	3.78	3.12	3.60	3.42	3.26	2.84	3.33	3.29

	Have you been vaccinated against COVID-19 (coronavirus)?			
	I have not been vaccinated	I have been vaccinated once	I have been vaccinated more than once	I don’t remember
%				
(1) Definitely not	19	10	17	8
(2) Somewhat not	**4−**	14	**22+ **	6
(3) Hard to say	**9−**	11	**22+ **	16
(4) Somewhat yes	**5−**	19	**17+ **	**–**
(5) Definitely not	**63+ **	46	**23−**	**70+ **
(1 + 2) NO	**23−**	24	**39+ **	15
(4 + 5) YES	**69+ **	**65+ **	**40−**	70
Total	100	100	100	100
Mean	3.90	3.76	3.07	4.16

In Poland, people with health insurance (the vast majority of the population) have the right to a free hospital stay. However, during the COVID-19 pandemic, when hospitals were busy and overcrowded, an idea appeared in a public debate that people who avoided vaccinations should bear the costs of hospital stays if they contracted the coronavirus. However, our research results show that in Poland, where people are strongly accustomed to free healthcare, only 18% supported this idea, and as many as 69% (including 46% who supported it strongly) were against the introduction of fees for hospital stays (Table 5. The greatest number of opponents of this solution were among supporters of the right-wing populist Confederation (85%) and unvaccinated people (88%). (Table 5). However, the issue of financing the consequences of risky behaviour (such as avoiding vaccinations) by all taxpayers requires public debate. Just as committing road accidents and offences that threaten the safety of other people results in an increase in civil insurance premiums for drivers in Poland, risky behaviour towards public health and the safety of other people also requires debate and the development of appropriate legal regulations. Risky and selfish health behaviours in the public sphere resembling fare evasion should not be accepted.

**Table 5:** A few years after the outbreak of the pandemic and the widespread vaccination campaign against COVID-19, we have more knowledge on this subject. Do you agree with the following statement: ‘Unvaccinated people should cover the costs of their hospital stay if they become infected’? (In percentage)

	Sex			Age						Education			
	Total	Male	Female	18–24	25–29	30–39	40–49	50–59	60+	Primary or middle	Vocational	Secondary	Higher
%													
(1) Definitely not	46	47	44	55	46	53	**61+ **	38	**33−**	41	42	**52+ **	43
(2) Somewhat not	23	**18−**	**28+ **	21	28	27	17	18	26	23	25	21	24
(3) Hard to say	13	12	13	15	11	**5−**	11	20	15	18	11	12	13
(4) Somewhat yes	9	**13+ **	**6−**	8	6	**6−**	7	12	**14+ **	6	**15+ **	8	9
(5) Definitely yes	9	9	9	**2−**	9	9	**5−**	13	12	12	7	7	11
(1 + 2) NO	69	65	72	76	75	**80+ **	**77+ **	**56−**	**59−**	64	67	**73+ **	66
(4 + 5) YES	18	**22+ **	**15−**	**9−**	14	15	**11−**	25	**25+ **	19	21	15	20
Total	100	100	100	100	100	100	100	100	100	100	100	100	100
Mean	2.13	2.19	2.07	1.80	2.02	1.91	1.78	2.44	2.45	2.26	2.18	1.97	2.22

	Political sympathies									How often do you attend masses, church services or religious meetings?						
	Law and Justice	The Third Way: Poland Szymon Hołownia’s Poland 2050 and Polish People’s Party	Civic Coalition	New Left	Confederation	Nonpartisan Local Government Activists	There is One Poland	Other	I don’t know, it’s hard to say	Every day or several times a week	Once a week every Sunday	Once or twice a month	Once or twice a year	Less than once a year	Never	Don’t know, I refuse to answer
%																
(1) Definitely not	42	46	**34−**	**26−**	**77+ **	54	**88+ **	54	46	**64+ **	44	50	48	42	41	44
(2) Somewhat not	27	21	22	**45+ **	**8−**	14	**4−**	40	20	15	26	21	21	25	21	26
(3) Hard to say	11	11	15	9	11	17	**–**	5	19	8	13	14	11	14	12	25
(4) Somewhat yes	10	14	11	10	**3−**	7	9	**–**	9	9	9	8	12	13	8	3
(5) Definitely yes	10	8	**18+ **	9	**0−**	8	**–**	**–**	7	4	8	6	8	6	**17+ **	**2−**
(1 + 2) NO	69	67	**56−**	71	**85+ **	68	91	**95+ **	66	79	70	72	69	67	63	70
(4 + 5) YES	20	22	**29+ **	20	**4−**	15	9	**–**	16	13	17	14	20	19	**25+ **	**5−**
Total	100	100	100	100	100	100	100	100	100	100	100	100	100	100	100	100
Mean	2.20	2.16	2.56	2.32	1.42	2.02	1.30	1.51	2.11	1.73	2.12	1.98	2.10	2.16	2.38	1.94

	Have you been vaccinated against COVID-19 (coronavirus)?			
	I have not been vaccinated	I have been vaccinated once	I have been vaccinated more than once	I don’t remember
%				
(1) Definitely not	**78+ **	**59+ **	**30−**	**75+ **
(2) Somewhat not	**10−**	27	**28+ **	**–**
(3) Hard to say	**5−**	**6−**	**17+ **	7
(4) Somewhat yes	**5−**	6	**12+ **	**–**
(5) Definitely yes	**1−**	**2−**	**13+ **	18
(1 + 2) NO	**88+ **	**86+ **	**58−**	75
(4 + 5) YES	**6−**	**8−**	**25+ **	18
Total	100	100	100	100
Mean	1.41	1.65	2.49	1.85

### Organization of vaccinations and institutions responsible for vaccine policy

As the research results show, most Poles share the view that the organization of vaccinations should be handled by state healthcare centres (hospitals, public clinics). Overall, 68% of respondents supported this idea. This view not only indicates attachment to public health service centres, but is also a manifestation of trust in public health institutions, which, despite many flaws, give the impression of being the most professional centres when it comes to vaccinations. This trust can be strengthened by an appropriate information policy and clear provision of knowledge about all vaccination measures and effects [55]. This is an important issue because the level of trust in public health institutions has a significant impact on vaccination practices. In the United States, hesitancy and negative perceptions of COVID-19 vaccines affected trust in public health institutions [56].

Supporters of the view that private health centres offering paid services should be responsible for vaccinations constituted a distinct minority (4%). The idea that local authorities, municipal institutions or schools should organize vaccinations was also not appreciated (4%). The only idea against the organization of vaccinations by state institutions that was also disseminated by the anti-vaccination movements and was often indicated by respondents was that “no one should have the right to organize mass and compulsory vaccinations” (19%) (Table 6). This view of nearly 20% of the population reflects the real number of hard-line opponents of vaccinations in Poland. Most of them were among supporters of the populist right (nearly 50%) and those who were not vaccinated against COVID-19 at all (39%). It is worth noting that the strongest anti-vaxxers were in the age category of 30–39 years.

**Table 6:** Who should be responsible for organizing vaccinations on a daily basis? (In percentage)

	Sex			Age						Education			
	Total	Male	Female	18–24	25–29	30–39	40–49	50–59	60+	Primary or middle	Vocational	Secondary	Higher
%													
Private clinics and private health centres where everyone pays for the services they want to use	4	5	4	8	5	6	3	4	3	2	4	5	4
State healthcare centres (hospitals, public clinics)	68	**64−**	**72+ **	**52−**	65	**60−**	65	76	**77+ **	59	69	69	71
Local authorities, municipal institutions, schools and kindergartens	4	4	3	11	5	4	3	1	2	4	2	4	4
No one should have the right to organize mass and mandatory vaccinations	19	21	18	28	23	**28+ **	25	17	**9−**	20	22	18	18
I don’t know/hard to say	5	6	4	**1−**	2	**2−**	5	3	**9+ **	15	3	3	3
Total	100	100	100	100	100	100	100	100	100	100	100	100	100

	Political sympathies									How often do you attend masses, church services or religious meetings?						
	Law and Justice	The Third Way: Poland Szymon Hołownia’s Poland 2050 and Polish People’s Party	Civic Coalition	New Left	Confederation	Nonpartisan Local Government Activists	There is One Poland	Other	I don’t know, it’s hard to say	Every day or several times a week	Once a week every Sunday	Once or twice a month	Once or twice a year	Less than once a year	Never	Don’t know, I refuse to answer
%																
Private clinics and private health centres where everyone pays for the services they want to use	3	5	4	**–**	11	3	4	5	2	4	5	6	2	5	4	6
State healthcare centres (hospitals, public clinics)	**78+ **	70	**78+ **	**86+ **	**36−**	**46−**	84	61	66	78	73	65	73	57	**59−**	**87+ **
Local authorities, municipal institutions, schools and kindergartens	**1−**	4	6	3	11	**–**	**–**	**–**	1	2	2	2	3	9	6	**–**
No one should have the right to organize mass and mandatory vaccinations	15	14	**9−**	10	**43+ **	**51+ **	12	17	28	12	15	25	19	26	22	**5−**
I don’t know/hard to say	2	7	3	**–**	**–**	**–**	**–**	18	3	3	5	3	2	3	**9+ **	2
Total	100	100	100	100	100	100	100	100	100	100	100	100	100	100	100	100

	Have you been vaccinated against COVID-19 (coronavirus)?			
	I have not been vaccinated	I have been vaccinated once	I have been vaccinated more than once	I don’t remember
%				
Private clinics and private health centres where everyone pays for the services they want to use	**8+ **	4	**3−**	13
State healthcare centres (hospitals, public clinics)	**42−**	**55−**	**80+ **	67
Local authorities, municipal institutions, schools and kindergartens	3	6	4	**–**
No one should have the right to organize mass and mandatory vaccinations	**39+ **	28	**10−**	20
I don’t know/hard to say	8	8	**3−**	**–**
Total	100	100	100	100

### Voluntary versus mandatory vaccinations

Questions about voluntary versus mandatory vaccinations and the debate on this issue take place not only in the media [57], but also in scientific journals. According to Alberto Giubilini:

Vaccine refusal is morally equivalent to tax evasion and should be legally treated like tax evasion. This means that non-vaccination should be illegal, except in cases where there are medical reasons for not vaccinating [58].

From the perspective of common knowledge, the matter becomes more complicated in the case of childhood vaccinations. However, in the face of the decreasing number of vaccinations (e.g. against measles), there are voices in the debate that vaccination of children should not be seen as part of the parents’ choice, but as a non-negotiable legal obligation. According to Roland Pierik, “first, government should not permit parents to put their children at avoidable risk of death and suffering; second, government should guard the common good of herd immunity to protect vulnerable persons” [59]. During the COVID-19 pandemic, when the risk of the disease for children was still unknown, there was a debate about the compulsory vaccination of children against influenza. It was stated that “mandatory vaccination of children for influenza with mild to moderate coercion could be justified. This practice might include reasonably onerous opt-out procedures or perhaps modest fines” [60] (p. 14). Do these arguments influence social attitudes? In our research, there were two options for mandatory vaccinations: “mandatory up to the age of 18, and during the epidemic, mandatory in all age categories” and “mandatory up to the age of 18, and completely voluntary in later adulthood”. These were chosen by 15% and 12% of respondents, respectively. As regards the first option, results higher than 15% applied to liberal (26%) and leftist (38%) electorates, those vaccinated against the coronavirus at least twice (22%) and atheists (people who had never participated in religious practices) (22%) (Table 7). The view that gained the most support in society as a whole was that vaccinations should be “completely voluntary and anyone interested should have access to them at any time” (47%).

**Table 7:** Do you think that vaccinations should be voluntary or mandatory? (In percentage)

	Sex			Age						Education			
	Total	Male	Female	18–24	25–29	30–39	40–49	50–59	60+	Primary or middle	Vocational	Secondary	Higher
%													
They should be completely voluntary and anyone interested should have access to them at any time	47	46	48	**27−**	**30−**	53	42	51	**54+ **	58	53	47	**36−**
They should be mandatory up to the age of 18 and during epidemics in all age categories	15	14	15	20	21	**9−**	10	15	18	11	12	14	**19+ **
The state should not impose the obligation to vaccinate anyone, and only parents can decide whether to vaccinate their children	24	23	25	33	25	24	**33+ **	22	**17−**	19	28	22	25
Mandatory up to the age of 18, and completely voluntary in later adulthood	12	13	11	20	22	10	12	10	9	10	**6−**	14	15
I don’t know/hard to say	3	4	1	**–**	2	3	4	2	3	2	1	2	4
Total	100	100	100	100	100	100	100	100	100	100	100	100	100

	Political sympathies									How often do you attend masses, church services or religious meetings?						
	Law and Justice	The Third Way: Poland Szymon Hołownia’s Poland 2050 and Polish People’s Party	Civic Coalition	New Left	Confederation	Nonpartisan Local Government Activists	There is One Poland	Other	I don’t know, it’s hard to say	Every day or several times a week	Once a week every Sunday	Once or twice a month	Once or twice a year	Less than once a year	Never	Don’t know, I refuse to answer
%																
They should be completely voluntary and anyone interested should have access to them at any time	**57+ **	42	39	**24−**	34	65	34	34	**60+ **	45	**57+ **	44	42	42	**37−**	63
They should be mandatory up to the age of 18 and during epidemics in all age categories	**8−**	16	**26+ **	**38+ **	**–**	12	**–**	22	**7−**	**5−**	11	10	18	15	**22+ **	13
The state should not impose the obligation to vaccinate anyone, and only parents can decide whether to vaccinate their children	25	22	19	17	**41+ **	17	55	17	21	35	20	**33+ **	25	20	24	16
Mandatory up to the age of 18, and completely voluntary in later adulthood	**8−**	19	13	16	22	6	11	11	9	12	**8−**	12	13	20	15	5
I don’t know/hard to say	2	1	2	4	3	**–**	**–**	16	3	3	4	1	2	3	2	3
Total	100	100	100	100	100	100	100	100	100	100	100	100	100	100	100	100

	Have you been vaccinated against COVID-19 (coronavirus)?			
	I have not been vaccinated	I have been vaccinated once	I have been vaccinated more than once	I don’t remember
%				
They should be completely voluntary and anyone interested should have access to them at any time	48	54	46	34
They should be mandatory up to the age of 18 and during epidemics in all age categories	**1−**	**5−**	**22+ **	18
The state should not impose the obligation to vaccinate anyone, and only parents can decide whether to vaccinate their children	**40 + **	28	**17−**	37
Mandatory up to the age of 18, and completely voluntary in later adulthood	8	10	**14+ **	**–**
I don’t know/hard to say	3	4	2	12
Total	100	100	100	100

However, the statement that is also disseminated by anti-vaxxers that “the state should not impose the obligation to vaccinate anyone, and only parents can decide whether to vaccinate their children” was chosen by 24% of respondents. Again, it was the most popular among Confederation sympathizers (41%), people not vaccinated against COVID-19 (40%) and those who regularly attended religious services once a week (33%). The latter variable may indicate that more religious people who support the traditional family model also defend a conservative position on vaccinations, in which the family, and not the state or another actor of institutionalized public life, should decide whether to vaccinate their children.

### Health education and information policy about vaccinations

Clear information provided in a language understandable to people with different educational backgrounds is an important tool for health education and a means of overcoming stereotypes about vaccinations [61]. Education can particularly influence parents’ decisions about vaccinating their children [62]. Hence, society’s views on the health education process and who can be trusted are very important. In Polish society, the state and its institutions were most often mentioned (52%) as the actors responsible for health education and dissemination of information about the importance and effects of vaccines. However, in second place was “citizen research and scientific institutions and social organizations working for public health” (26%) (Table 8). However, this result may be misleading. Although this topic requires further research and a separate article, it can be said here that “civil society” can gather both progressive organizations and those that undermine trust in the science and traditions of the Enlightenment. From 2015 to 2023, the right-wing populist government in Poland centralized the process of distributing funds to civil society, clearly preferring conservative and nationalist organizations. In this context, people even talked about “the dark side of civil society” [63]. It certainly also includes nationalist, populist and anti-scientific anti-vaccination movements. Hence, the category of people who support the view that “social organizations working for public health should deal with medical education” includes both supporters of the democratization of healthcare and greater civic participation in and supervision over vaccination policy and those who support conspiracy theories and anti-Enlightenment myths. This view is confirmed by the relatively high percentage of supporters of the populist Confederation who chose this option (41%).

**Table 8:** Who should be responsible for health education and dissemination of information about the importance and effects of vaccines? (In percentage)

	Sex			Age						Education			
	Total	Male	Female	18–24	25–29	30–39	40–49	50–59	60+	Primary or middle	Vocational	Secondary	Higher
%													
Anyone interested should educate themselves	12	12	13	9	11	15	14	9	13	21	15	**9−**	10
Parents	6	6	5	8	**1−**	6	4	7	7	5	**11+ **	5	4
The state and its institutions	52	55	49	**34−**	60	47	49	**61+ **	55	40	46	55	**58+ **
Civic research and scientific institutions and social organizations working for the public health sector	26	23	28	**45+ **	27	29	28	22	**18−**	22	25	27	26
I don’t know/hard to say	5	4	6	4	**1−**	2	5	**1−**	**8+ **	12	3	5	**2−**
Total	100	100	100	100	100	100	100	100	100	100	100	100	100

	Political sympathies									How often do you attend masses, church services or religious meetings?						
	Law and Justice	The Third Way: Poland Szymon Hołownia’s Poland 2050 and Polish People’s Party	Civic Coalition	New Left	Confederation	Nonpartisan Local Government Activists	There is One Poland	Other	I don’t know, it’s hard to say	Every day or several times a week	Once a week every Sunday	Once or twice a month	Once or twice a year	Less than once a year	Never	Don’t know, I refuse to answer
%																
Anyone interested should educate themselves	15	9	**6−**	6	11	18	14	13	12	13	16	17	**8−**	7	13	6
Parents	9	**3−**	**2−**	3	13	7	7	**–**	11	12	6	9	4	4	4	11
The state and its institutions	50	55	**72+ **	**69+ **	**30−**	48	23	61	50	39	52	**41−**	**60+ **	53	52	53
Civic research and scientific institutions and social organizations working for the public health sector	22	28	**19−**	23	**41+ **	25	52	20	25	33	23	30	26	31	23	24
I don’t know/hard to say	4	6	**1−**	**–**	5	3	4	6	3	3	4	4	2	5	8	6
Total	100	100	100	100	100	100	100	100	100	100	100	100	100	100	100	100

	Have you been vaccinated against COVID-19 (coronavirus)?			
	I have not been vaccinated	I have been vaccinated once	I have been vaccinated more than once	I don’t remember
%				
Anyone interested should educate themselves	**19+ **	16	**9−**	**–**
Parents	9	5	5	10
The state and its institutions	**32−**	**39−**	**61+ **	29
Civic research and scientific institutions and social organizations working for the public health sector	**34+ **	32	**21−**	41
I don’t know/hard to say	6	8	3	20
Total	100	100	100	100

### Financing, fees for vaccinations and organization of research on new vaccines

Access to vaccinations should always be free everywhere according to the vast majority of Polish society (65%), or free for children and adolescents up to 18 years of age and for all age categories during epidemics (23%) (Table 9). These results prove that Polish society believes the state should participate in developing health policy and financing healthcare and vaccinations. Although the costs of vaccines are not high throughout life [64], individuals may assess these expenses differently depending on their social position. The results obtained, and particularly the position of supporters of the populist Confederation, indicate that those with the hesitant orientation share the neoliberal view that “vaccinations should be widely available, but paid for by interested individuals”. Only 7% of the general public, but as many as 24% of Confederation supporters, believed so.

**Table 9:** Who do you think should finance the organization of vaccinations? (In percentage)

	Sex			Age						Education			
	Total	Male	Female	18–24	25–29	30–39	40–49	50–59	60+	Primary or middle	Vocational	Secondary	Higher
%													
Vaccinations should be widely available, but paid for by the individuals concerned	7	**10+ **	**5−**	15	14	9	8	7	**2−**	11	4	7	8
Vaccinations should be paid and should not be widely available (for example, they should be available on prescription)	2	3	2	4	1	3	3	2	**0−**	**–**	2	2	4
Vaccinations should be free for children and adolescents up to 18 years of age and during epidemics for all age categories	23	22	24	27	24	29	22	23	18	19	17	24	**28+ **
All vaccinations should be free and financed from universal health insurance contributions	65	64	67	54	61	**56−**	64	65	**75+ **	67	**73+ **	63	**59−**
I don’t know/hard to say	2	2	3	**–**	**–**	2	2	3	4	3	3	3	**1−**
Total	100	100	100	100	100	100	100	100	100	100	100	100	100

	Political sympathies									How often do you attend masses, church services or religious meetings?						
	Law and Justice	The Third Way: Poland Szymon Hołownia’s Poland 2050 and Polish People’s Party	Civic Coalition	New Left	Confederation	Nonpartisan Local Government Activists	There is One Poland	Other	I don’t know, it’s hard to say	Every day or several times a week	Once a week every Sunday	Once or twice a month	Once or twice a year	Less than once a year	Never	Don’t know, I refuse to answer
%																
Vaccinations should be widely available, but paid for by the individuals concerned	5	6	4	**1−**	**24+ **	12	11	4	11	15	6	12	6	10	6	2
Vaccinations should be paid and should not be widely available (for example, they should be available on prescription)	1	**0−**	1	**–**	**10+ **	16	6	**–**	1	6	1	4	3	1	1	3
Vaccinations should be free for children and adolescents up to 18 years of age and during epidemics for all age categories	**16−**	31	23	26	17	15	18	40	24	18	21	22	29	15	27	14
All vaccinations should be free and financed from universal health insurance contributions	**74+ **	62	70	74	**45−**	58	65	56	60	56	70	60	61	72	61	74
I don’t know/hard to say	3	1	1	**–**	3	**–**	**–**	**–**	4	5	1	2	2	2	4	6
Total	100	100	100	100	100	100	100	100	100	100	100	100	100	100	100	100

	Have you been vaccinated against COVID-19 (coronavirus)?			
	I have not been vaccinated	I have been vaccinated once	I have been vaccinated more than once	I don’t remember
%				
Vaccinations should be widely available, but paid for by the individuals concerned	**16+ **	10	**4−**	**–**
Vaccinations should be paid and should not be widely available (for example, they should be available on prescription)	**6+ **	2	**1−**	4
Vaccinations should be free for children and adolescents up to 18 years of age and during epidemics for all age categories	20	24	24	**–**
All vaccinations should be free and financed from universal health insurance contributions	**53−**	62	**70+ **	**93+ **
I don’t know/hard to say	5	3	**1−**	4
Total	100	100	100	100

However, when it comes to deciding who should conduct research on new vaccines, the most popular answers were “the state and private companies supervised by public opinion, civic research institutes and social organizations” (42%) and “only the state and its subordinate institutions” (38%) (Table 10). It is worth noting that the first option was chosen particularly often by young people aged 25–29 years (72%) and those aged 30–39 years (53%), as well as supporters of the left (59%) and people with higher education (60%) (Table 10). This indicates that among vaccination supporters there is also a strong longing for democratization, socialization and giving a more civic character to research on new vaccines and treatment of people. This group certainly also includes those who are critical of the activities of large pharmaceutical companies, and driven more by the idea of social justice than opposition to science, would like greater supervision over this market sector.

**Table 10:** Who should conduct research on new vaccines and new methods of treating people? (In percentage)

	Sex			Age						Education			
	Total	Male	Female	18–24	25–29	30–39	40–49	50–59	60+	Primary or middle	Vocational	Secondary	Higher
%													
Private companies and concerns	5	7	4	**16+ **	6	5	8	3	**3−**	4	6	6	5
The state, taking into account the will of the patient’s family	10	10	10	14	**2−**	13	12	14	**7−**	15	13	8	8
Only the state and its subordinate institutions	38	38	37	**16−**	**17−**	**26−**	39	39	**54+ **	**53+ **	**52+ **	**32−**	**24−**
The state and private companies supervised by public, citizen research institutes and community healthcare organizations	42	42	42	51	**72+ **	**53+ **	38	41	**28−**	**17−**	**25−**	**49+ **	**60+ **
I don’t know/hard to say	5	3	7	4	4	**2−**	4	3	**9+ **	11	4	4	3
Total	100	100	100	100	100	100	100	100	100	100	100	100	100

	Political sympathies									How often do you attend masses, church services or religious meetings?						
	Law and Justice	The Third Way: Poland Szymon Hołownia’s Poland 2050 and Polish People’s Party	Civic Coalition	New Left	Confederation	Nonpartisan Local Government Activists	There is One Poland	Other	I don’t know, it’s hard to say	Every day or several times a week	Once a week every Sunday	Once or twice a month	Once or twice a year	Less than once a year	Never	Don’t know, I refuse to answer
%																
Private companies and concerns	4	5	4	3	**18+ **	12	3	**–**	3	**1−**	4	5	8	11	3	7
The state, taking into account the will of the patient’s family	11	10	7	**4−**	8	14	**57+ **	9	9	6	14	8	7	7	12	8
Only the state and its subordinate institutions	**51+ **	30	36	33	**23−**	34	23	62	38	38	**47+ **	30	38	30	**29−**	56
The state and private companies supervised by public, citizen research institutes and community healthcare organizations	**30−**	52	50	**59+ **	48	40	**13−**	26	44	48	**29−**	**52+ **	44	50	**51+ **	**23−**
I don’t know/hard to say	5	3	3	1	3	**–**	4	4	6	7	6	5	3	2	6	6
Total	100	100	100	100	100	100	100	100	100	100	100	100	100	100	100	100

	Have you been vaccinated against COVID-19 (coronavirus)?			
	I have not been vaccinated	I have been vaccinated once	I have been vaccinated more than once	I don’t remember
%				
Private companies and concerns	7	11	4	**–**
The state, taking into account the will of the patient’s family	**18+ **	**4−**	**8−**	**–**
Only the state and its subordinate institutions	33	30	**41+ **	20
The state and private companies supervised by public, citizen research institutes and community healthcare organizations	38	51	42	56
I don’t know/hard to say	3	4	5	24
Total	100	100	100	100

### Socio-demographic factors influencing attitudes towards vaccination practices in Poland

More than 4 years after the outbreak of the pandemic, there are still new SARS-CoV-2 infections, and long-COVID remains a major public health problem [65]. In these conditions, considerations about factors that influence preferences for the individual models of vaccination policy have not only cognitive, but also practical value.

First, we checked whether there were correlations between the three variables that were considered in the regression analysis (political preferences, education and age). In all cases, the correlations were statistically significant, but its strengths were different.

As regards respondents’ political preferences (Table 11), the result of the chi-squared test is statistically significant, as there was a relationship between political preferences and the level of vaccination. The variables were correlated with one another at a medium level. It is worth noting that – as has already been shown – while political preferences significantly affect attitudes towards vaccinations, they play a particularly large role among supporters of political groups with anti-elite world views [66]. In Polish conditions, these were voters of the Confederation and two extra-parliamentary groups: Nonpartisan Local Government Activists and There is One Poland.

**Table 11:** Cross-tabulation: vaccination and political sympathies

Have you been vaccinated against COVID-19 (coronavirus)?		Which political parties or alliances would you vote for?							
		Law and justice	The Third Way, that is, Szymon Hołownia’s Poland 2050 and Polish People’s Party	Civic coalition	New Left	Confederation	Nonpartisan Local Government Activists	There is One Poland	Total
I have not been vaccinated	% political sympathies	22.8	21.3	11.7	5.6	59.2	36.0	78.9	23.7
I have been vaccinated once	% political sympathies	5.9	8.5	8.3	9.9	17.1	4.0	0.0	8.5
I have been vaccinated more than once	% political sympathies	71.2	70.2	79.0	84.5	23.7	56.0	15.8	66.7
I don’t remember	% political sympathies	0.0	0.0	1.0	0.0	0.0	4.0	5.3	1.0
Total	% political sympathies	100.0	100.0	100.0	100.0	100.0	100.0	100.0	100.0

Chi-squared tests			
	Value	*df*	Asymptotic (two-sided) significance
Pearson’s chi-squared test	167.509	24	0.000
Credibility quotient	159.329	24	0.000
Linear correlation test	11.828	1	0.001
*N* important observations	859		

Symmetric measures			
		Value	Approximate significance
Nominal by nominal	Phi	0.442	0.000
	Cramer’s V	0.255	0.000
*N* important observations		859	

In studies on vaccination behaviour, the education variable is often mentioned as one of the important factors shaping attitudes towards vaccinations [67]. Our results confirmed this as well (Table 12), but the strength of the correlation was not statistically significant.

**Table 12:** Cross-tabulation: vaccination and education

Have you been vaccinated against COVID-19 (coronavirus)?	Education				Total
	Elementary and middle school	Basic vocational	Secondary	Higher	
I have not been vaccinated	32.6	27.0	26.2	19.9	25.6
I have been vaccinated once	2.8	9.4	11.4	8.7	9.0
I have been vaccinated more than once	64.5	62.7	61.0	70.3	64.4
I don’t remember	0.0	0.9	1.4	1.1	1.0
Total	100.0	100.0	100.0	100.0	100.0

Chi-squared tests			
	Value	*df*	Asymptotic (two-sided) significance
Pearson’s chi-squared test	19.058	9	0.025
Credibility quotient	22.432	9	0.008
Linear correlation test	5.482	1	0.019
*N* important observations	1001		

Symmetric measures					
		Value	Asymptotic standard error^a^	Approximate T^b^	Approximate significance
Nominal by nominal	Phi	0.138			0.025
	Cramer’s V	0.080			0.025
Interval by interval	Pearson’s *R*	0.074	0.032	2.347	0.019^c^
Ordinal by ordinal	Spearman’s correlation	0.069	0.031	2.200	0.028^c^
*N* important observations		1001			

^a^Without assuming the null hypothesis

^b^The asymptotic standard error was used, assuming the null hypothesis

^c^On the basis of the normal distribution approximation

It turned out that in the case of COVID-19, the variable of respondents’ age was more important in Poland. This correlation was statistically significant and the age factor had a greater impact on the vaccination practices of respondents than the variable of education (Table 13).

**Table 13:** Cross-tabulation: vaccination and age

Have you been vaccinated against COVID-19 (coronavirus)?	Declared age						Total
	18–24	25–29	30–39	40–49	50–59	60+	
I have not been vaccinated	36.9	21.1	28.9	40.3	21.8	14.4	25.5
I have been vaccinated once	13.1	15.8	14.9	6.5	6.8	5.1	9.0
I have been vaccinated more than once	50.0	60.5	55.2	52.7	70.1	79.8	64.6
I don’t remember	0.0	2.6	1.0	0.5	1.4	0.6	0.9
Total	100.0	100.0	100.0	100.0	100.0	100.0	100.0

Chi-squared tests			
	Value	*df*	Asymptotic (two-sided) significance
Pearson’s chi-squared test	83.696^a^	15	0.000
Credibility quotient	82.407	15	0.000
Linear correlation test	34.610	1	0.000
*N* important observations	999		

Symmetric measures					
		Value	Asymptotic standard error^a^	Approximate T^b^	Approximate significance
Nominal by nominal	Phi	0.289			0.000
	Cramer’s V	0.167			0.000
Interval by interval	Pearson’s *R*	0.186	0.030	5.985	0.000^c^
Ordinal by ordinal	Spearman’s correlation	0.202	0.030	6.514	0.000^c^
*N* important observations		999			

^a^Without assuming the null hypothesis

^b^The asymptotic standard error was used, assuming the null hypothesis

^c^On the basis of the normal distribution approximation

Taking into account the regression analysis with only education and age, these variables describe the variability of the vaccination level at 5.4%. Both coefficients were statistically significant. However, age had a greater impact on the vaccination level than education (Table 14).

**Table 14:** Regression analysis

Model – summary				
Model	*R*	*R*-squared	Adjusted *R*-squared	Standard error of estimate
1	0.232	0.054	0.052	0.856

Ananylsis of variance (ANOVA)						
Model		Root mean square	*df*	Root mean square	*F*	Significance
1	Regression	41.511	2	20.756	28.316	0.000
	Rest	730.795	997	0.733		
	Total	772.306	999			

Coefficients						
Model		Unstandardized coefficients		Standardized coefficients	*t*	Significance
		B	Standard error	Beta		
1	(Constant)	1.553	0.123		12.637	0.000
	Age	0.123	0.017	0.228	7.097	0.000
	Education	0.123	0.028	0.141	4.389	0.000

## Conclusions

The data obtained confirm the need to integrate theoretical findings about vaccination policy, facilitating the translation of knowledge into practice [68] and understanding what factors are important in a given country, social class and environment for making decisions about vaccination. The literature usually emphasizes various psychological factors relating to an individual. This article has shown that, from a sociological perspective, many important variables shape attitudes towards vaccination policy. At the macro-social level, political sympathies, cultural capital and education are certainly important, followed by the previous vaccination practices of certain people and communities. This article has shown that attitudes towards vaccinations are embedded in broader divisions and orientations related to the vision of the social order: the role of the state, the organization of healthcare and payments for vaccinations and medical services, as well as preferred ways of knowledge production in society and work on new vaccines. The authors show that the apparent anti-systemic nature of “anti-vaccination movements” can be weakened and unmasked by greater democratization of vaccination policy (allowing representatives of social organizations dealing with healthcare to participate in consultation and decision-making procedures in vaccination policy). The possible role of the health civil society sector in the vaccination process and public health in general requires separate and additional analyses.

The answers obtained to the survey questions indicate that the number of people reluctant to vaccinate in Poland varies, depending on the context, from approximately 20% (the most dogmatic opponents of vaccination) to approximately 30% (those with greater or lesser doubts about the use of vaccinations). It would be worth carrying out comparative analyses in different societies to indicate how cultural differences, economic models, healthcare models and political systems affect the percentage of anti-vaxxers in different countries. This article also encourages further debate on the issue of mandatory vaccinations and the costs of treating infectious diseases for people who consciously refuse vaccinations. Research on the relationship between the type and role of education and attitudes towards vaccinations would be equally important and helpful in Poland [69]. It is worth studying whether it is higher education or rather a specific type of higher education (e.g. relations between respondents with higher education in the humanities, technology and medicine) that has such a great influence.

A limitation of this research is the lack of migrants and refugees from Ukraine in the research sample. They constitute more than a million inhabitants of Poland approximately, and during the first weeks of the war in Ukraine in the spring of 2022, the number of refugees reached 4,137,842 people [70]. This undoubtedly affected the epidemiological situation, especially since a large number of adult immigrants from Ukraine did not complete official vaccination programmes and presented a rather specific approach to vaccinations [e.g. they had no knowledge of human papillomavirus (HPV) vaccinations and were sceptical about influenza vaccinations] [71]. However, this was not taken into account in our research.

In addition, it is worth emphasizing that the vaccination policy preferences described in this article may be part of a larger phenomenon, which is trust in and attitude towards scientific knowledge (not only vaccinations but also climate change and green transition goals, etc.) [72]. However, this is material for a separate article.

## Data Availability

The datasets used and/or analysed during the current study are available from the corresponding author on reasonable request.
